# Impairment of Macrophage Functions by the Senescence-Associated Secretory Phenotype of Vascular Smooth Muscle Cells—Brief Report

**DOI:** 10.1161/ATVBAHA.125.324299

**Published:** 2026-04-02

**Authors:** Dimitrios Tsitsipatis, Tatiana Rodriguez Rivera, Mary Kaileh, Ada N. Okereke, Aditi Gupta, Amit Singh, Sean M. Raph, Charnae’ Henry-Smith, Allison B. Herman

**Affiliations:** 1 Laboratory of Cardiovascular Science (D.T., T.R.R., A.N.O., S.M.R., C.H.-S., A.B.H.), National Institute on Aging (NIA) Intramural Research Program (IRP), National Institutes of Health (NIH), Baltimore, MD.; 2 Laboratory of Molecular Biology and Immunology (M.K., A.G., A.S.), National Institute on Aging (NIA) Intramural Research Program (IRP), National Institutes of Health (NIH), Baltimore, MD.

**Keywords:** culture media, conditioned, efferocytosis, foam cells, macrophages, senescence-associated secretory phenotype

## Abstract

**BACKGROUND:**

This study aimed to determine the effect of senescent vascular smooth muscle cells (VSMCs) on foam cell formation and macrophage phagocytic activity in atherosclerotic conditions.

**METHODS:**

We measured the capacity of senescent VSMCs for scavenging oxLDL (oxidized low-density lipoprotein), which was impaired in senescent cells compared with proliferating and quiescent cells. Next, we obtained human peripheral blood monocytes from people >60 years old and differentiated them into macrophages using GM-CSF (granulocyte-macrophage colony-stimulating factor). We treated the macrophages with conditioned media derived from proliferating, quiescent, and senescent VSMCs and measured oxLDL uptake, phagocytosis, and efferocytosis.

**RESULTS:**

The results demonstrated that macrophages treated with senescent VSMC conditioned media experienced impaired oxLDL uptake, phagocytic activity, and reduced ability to clear senescent cells. Treatment of senescent VSMCs with senomorphic drugs before conditioned media transfer restored macrophage functions, confirming that the SASP (senescence-associated secretory phenotype) is critical for impairing macrophages during atherosclerotic conditions.

**CONCLUSIONS:**

Our results suggest that the SASP derived from senescent VSMCs prevents foam cell formation and disrupts the homeostatic function of macrophages in atherosclerosis. By suppressing macrophage function, senescent cells seem to evade immune clearance and accumulate, further propagating disease development.

**What Are the Clinical Implications?**

This study identifies a previously underappreciated functional consequence of vascular smooth muscle cell senescence, which is suppression of macrophage phagocytosis and efferocytosis through the SASP (senescence-associated secretory phenotype). We demonstrate that senescent vascular smooth muscle cells actively disrupt homeostatic macrophage behavior, impairing lipid uptake and clearance of senescent cells. These findings provide a mechanistic framework for how senescent vascular cells may evade immune surveillance and persist within the vessel wall. Importantly, senomorphic interventions across 3 pharmacologically distinct agents restored macrophage function, highlighting the therapeutic potential of targeting the SASP rather than eliminating cells outright. Although conducted under controlled experimental conditions, these results suggest that modulation of vascular senescence may represent a strategy to preserve macrophage homeostasis and limit maladaptive immune remodeling in age-related vascular disease. Future studies will determine how these mechanisms function in complex systems and whether SASP-targeted therapies can mitigate vascular dysfunction and improve clinical outcomes.

Cellular senescence occurs when a cell experiences sublethal stress and enters a state of persistent cell cycle arrest. Although in this state, senescent cells increase production of the SASP (senescence-associated secretory phenotype), increase levels of cell survival proteins, and elevate SA-β-Gal (senescence-associated β-galactosidase activity).^1^ Senescent cells have been implicated in disease and aging in both animal models and in the human vasculature.^2–4^ SASP factors are often the culprit of the detrimental function of senescence cells, as the secretion of cytokines and chemokines recruits and activates immune cells, promoting inflammation and oxidative stress in the vascular walls and contributing to the development of diseases, such as atherosclerosis. Senescent vascular smooth muscle cells (VSMCs) also aid in destabilizing atherosclerotic plaques by secreting inflammatory and hemostatic factors, among others.^4,5^ Importantly, senolytic drugs, which selectively remove senescent cells, have demonstrated strong efficacy in preclinical models of vascular aging and disease (reviewed extensively).^6^ A second class of drugs, termed senomorphics, directly suppresses the SASP and has shown the ability to improve disease and aging outcomes, suggesting an alternative avenue for senotherapies.

The immune system’s inability to clear these damaging cells is an understudied but critical aspect of senescent cell accumulation in the vasculature, as this is evident as we age and with disease, suggesting an imbalance of clearance during pathological conditions.^7,8^ We hypothesize that senescent cells escape immunosurveillance to promote immune and vascular cell dysfunction in the atherosclerotic niche. Macrophages are key players in the development of atherosclerosis. These phagocytic cells are critical scavengers of oxidized lipoproteins and cellular debris but often become overwhelmed during atherosclerosis, resulting in foam cell formation and subsequent cell death.^9^ Efferocytosis, the clearance of apoptotic or damaged cells, is significantly impaired during atherosclerosis, preventing the removal of those former foam cells, limiting reverse cholesterol transport, and facilitating the development of new foam cells in the plaque.^10^ These dysfunctional cellular processes contribute to plaque formation, destabilization, and likely interact with senescent VSMCs.

In this study, we found that senescent VSMCs have impaired oxLDL (oxidized low-density lipoprotein) uptake compared with quiescent and proliferating VSMCs. We also discovered that conditioned media (CM) from senescent VSMCs inhibits oxLDL uptake as well as phagocytosis in macrophages from human donors that were older than 60 years. We uncovered that lipopolysaccharide-stimulated human macrophages are effective at clearing senescent cells; however, treatment with CM from senescent VSMCs diminishes the clearance ability of macrophages, suggesting senescent VSMCs use paracrine signaling by SASP factors to suppress macrophage function and evade clearance. Lastly, we observed that treatment with SASP-suppressing senomorphic drugs rescued the ability of macrophages to uptake oxLDL and clear senescent cells, directly implicating the SASP as a driver of macrophage dysfunction.

## Materials and Methods

Detailed information about the resources used in this study is summarized in the Major Resources Table in the Supplemental Material.

### Data Availability

The data from this study are available from the corresponding author on reasonable request.

The mass spectrometry data are deposited in the MassIVE repository: (https://massive.ucsd.edu/ProteoSAFe/static/massive.jsp) with the data set identifier MSV000100792.

### Cell Culture, Macrophage Differentiation, Senescence Induction, SA-β-Galactosidase Activity, and Interleukin 8 and Tumor Necrosis Factor-α Secretion

Primary human coronary artery VSMCs from a 20-year-old donor were obtained as cryopreserved secondary cultures and maintained in VascuLife SMC Medium Complete Kit (VSMC media, LifeLine Cell Technology) at 37 °C in a humidified atmosphere according to the manufacturer’s protocol. Cells were serum starved for 3 days to promote quiescence, whereas senescence was established by treating once with 85 nM doxorubicin and replacing the media 3× for up to 10 days. Regarding the senomorphic treatments, proliferating and senescent VSMCs were treated with 10 mM of metformin, 100 nM of rapamycin, 1 μM of fisetin, or control vehicle (dimethyl sulfoxide) in serum-free media for 24 hours. Senescence-associated β-galactosidase (SA-β-gal) activity was assessed according to the manufacturer’s instructions (Cell Signaling). Quantification of the SA-β-gal signal was performed using ImageJ. For CM collection, proliferating, quiescent, and senescent cells were cultured for 24 hours in serum-free media or for 24 hours in serum-free media supplemented with senomorphic drugs in the senomorphic-treated proliferating and senescent cells. Secretion of IL (interleukin) 8 and TNF-α (tumor necrosis factor-α) was assessed using IL8 and TNF-α ELISAs (R&D Systems), respectively, according to the manufacturer’s instructions. To calculate relative secretion, we normalized the ELISA values to whole-cell protein extracts.

To enrich human monocytes, cryovials of human peripheral blood mononuclear cells were taken out of the liquid nitrogen freezer and immersed in a 37 °C water bath with stirring until the media was partially thawed. The cell suspension was then transferred to 9 mL of cold RPMI (Roswell Park Memorial Institute) 1640 media supplemented with 10% FBS and centrifuged at 456×*g* for 10 minutes at room temperature. Cells were resuspended in warm RPMI media at a concentration of 2 million peripheral blood mononuclear cells per mL and incubated at 37 °C for 1 hour in a humidified atmosphere. After incubation, cells were centrifuged at 456×*g*, counted, and resuspended at a concentration of 50 million peripheral blood mononuclear cells per 1 mL in Robosep buffer according to the manufacturer’s instructions. Monocytes were enriched from the peripheral blood mononuclear cells using an immunomagnetic negative selection EasySep human monocytes isolation kit (StemCell Technologies) using the automated cell separator RoboSep according to the manufacturer’s instructions (StemCell Technologies). Donors are volunteers who have donated apheresis packs through the Cytapheresis of Volunteer Donors protocol (National Institute on Aging protocol no. 03-AG-N316). Donors provided informed consent for their donations, and the protocol was approved by the institutional review board of the National Institute of Environmental Health Sciences of the National Institutes of Health. The demographics of the donors are available in Table S1.

For differentiating monocytes to macrophages, 1.5×10^6^ enriched monocytes were seeded in 2 mL of ImmunoCult-SF Macrophage Medium (macrophage media, StemCell Technologies) and differentiated to macrophages using 50 ng/mL of recombinant human GM-CSF (granulocyte-macrophage colony-stimulating factor; R&D Systems) for 7 days at 37 °C in a humidified atmosphere. Macrophages were then treated for 16 hours with CM (400 μL of CM in 1.2 mL of ImmunoCult-SF Macrophage Medium) derived from proliferating, quiescent, or senescent cells, as well as senomorphic-treated proliferating and senescent cells, and oxLDL uptake, phagocytosis activity, and efferocytosis were assessed as described below. We also treated the macrophages with macrophage media alone, a mixture of macrophage and VSMC media (400 μL of serum-free VSMC media in 1.2 mL of ImmunoCult-SF Macrophage Medium), and 100 ng/mL of lipopolysaccharide (Invitrogen) as controls; the lipopolysaccharide treatment was performed in macrophage media.

### OxLDL Uptake, Phagocytosis Activity, and Efferocytosis

#### OxLDL Uptake

For assessing oxLDL uptake in both VSMCs and macrophages, cells were incubated with 30 μg/mL of diI-oxLDL (1,1’-dioctadecyl-3,3,3’,3’-tetramethylindocarbocyanine perchlorate–oxLDL; Thermo Fisher Scientific) for 4 hours at 37 °C in a humidified atmosphere. For assessing oxLDL uptake in VSMCs, cells were incubated for 16 hours in serum-free media at 37 °C in a humidified atmosphere. Media was then aspirated, and the cells were washed once with the respective VSMC or macrophage media to remove excess oxLDL. Next, 1 mL of the respective fresh media was added, and oxLDL uptake was assessed on the Celigo (531/629 nm; Nexcelom Bioscience). Mean signal intensity was then normalized to the number of nuclei (377/447 nm).

#### Phagocytosis Activity

Macrophages were incubated with Green Zymosan particles (Abcam) for 1 hour at 37 °C in a humidified atmosphere. Media was then aspirated, and the cells were washed once with macrophage media to remove residual particles, and replaced with 1 mL of fresh macrophage media.

#### Efferocytosis by Macrophages

Proliferating and quiescent VSMCs were stained using PKH67 Green Fluorescent Cell Linker Kit for General Cell Membrane Labeling (Sigma-Aldrich), whereas senescent cells were stained using PKH26 Red Fluorescent Cell Linker Kit for General Cell Membrane Labeling (Sigma-Aldrich) for 2 minutes at room temperature in the dark. The stained VSMCs were then washed once with ice-cold PBS, resuspended in macrophage media to achieve a concentration of 3×10^5^ cells/1 mL, and immediately added to macrophages for a 2-hour incubation at 37 °C in a humidified atmosphere. We then aspirated the media, washed the cells once with fresh macrophage media, and added 1 mL of fresh macrophage media.

For all 3 assays, VSMCs and macrophages were incubated with NucBlue Live ReadyProbes Reagent (Hoechst 33342, Thermo Fisher Scientific) for 2 minutes at 37 °C in a humidified atmosphere before the final wash step; 3 to 4 images per condition per donor were taken using a fluorescence microscope (BZ-X Analyzer, Keyence).

### RNA Isolation and Reverse Transcription Followed by Quantitative Polymerase Chain Reaction Analysis

RNA was isolated using the TriPure isolation reagent (Roche) and the Direct-zol Mini Kit (Zymo Research), including a step of digestion with DNase, following the manufacturer’s protocol. Total RNA (500 ng) was reverse-transcribed into cDNA (complementary DNA) using Maxima reverse transcriptase (Thermo Fisher Scientific) and random hexamers and analyzed by quantitative polymerase chain reaction using SYBR Green mix (Kapa Biosystems). Relative RNA levels were calculated after normalizing to β-actin (*ACTB*) mRNA using the 2^−ΔΔCt^ method. The polymerase chain reaction primers used are listed in Table S2.

### Western Blot Analysis

Protein extracts were obtained by lysing cells with a denaturing buffer (2% sodium dodecyl sulfate [Sigma-Aldrich] in 50 mM HEPES). After boiling at 95 °C and sonication, 10 μg of whole-cell protein extracts were size-separated through polyacrylamide gels and transferred to nitrocellulose membranes (Bio-Rad). After transfer, membranes were blocked with 5% nonfat dry milk for 1 hour at room temperature and immunoprobed with the primary antibody overnight at 4 °C. Primary antibodies that recognized p16 (CDKN2A [cyclin-dependent kinase inhibitor 2A], sc-56330; Santa Cruz Biotechnology), GDF15 (growth differentiation factor 15; sc-377195; Santa Cruz Biotechnology), MCM2 (minichromosome maintenance complex component 2; 4007; Cell Signaling), phospho-NF-κB (nuclear factor-κB; S536; 3033; Cell Signaling), NF-κB (p65, 8242; Cell Signaling), phospho-mTOR (mechanistic target of rapamycin; S2481, Cell Signaling, 2974), mTOR (2983; Cell Signaling), phospho-AKT (protein kinase B; S473, 4060; Cell Signaling), AKT (4691; Cell Signaling), CD36 (cluster of differentation 36; ab133625; Abcam), CD206 (macrophage mannose receptor; ab64693; Abcam), and ACTB (sc-47778; Santa Cruz Biotechnology) were used. After a 1-hour incubation with the required secondary antibodies conjugated with horseradish peroxidase (Kindle Biosciences) at room temperature, the chemiluminescent signals were enhanced using KwikQuant Western blot detection kit (Kindle Biosciences) and detected on a Chemidoc system. To assess the relative CD36 and CD206 levels, densitometry analysis was performed using ImageJ.

### Sample Preparation for Mass Spectrometry and Proteomic Analysis

Samples were processed using the Proteograph (R) Product Suite.^11^ Briefly, 120 µL from 24 samples were transferred to the Seer Sample Preparation Plate for processing with the Proteograph ONE Assay kit (S55R5001; Seer Inc). Secreted proteins were quantitatively captured in nanoparticle-associated protein coronas. Proteins were subsequently denatured, reduced, alkylated, and subjected to proteolytic digestion (trypsin and Lys-C [endoproteinase Lysine C-terminus]). Peptides were purified, and peptide yields were determined using Pierce Quantitative Peptide Assays & Standards (Thermo Fisher Scientific). Peptides were then dried down overnight with a vacuum concentrator and reconstituted with a reconstitution buffer to a concentration of 35 ng/µL.

For data-independent acquisition (DIA), 8 µL of the reconstituted peptide mixture was analyzed, resulting in a constant 280 ng MS (mass spectrometry) injection per sample. Each sample was analyzed with a Vanquish NEO nanoLC system coupled with an Orbitrap Astral (Thermo Fisher) mass spectrometer using a trap-and-elute configuration. First, the peptides were loaded onto an Acclaim PepMap 100 C18 (0.3 mm ID×5 mm) trap column and then separated on a 50 cm µPACTM (micro-pillar array column; PharmaFluidics) at a flow rate of 1 µL/min using a gradient of 4% to 35% solvent B (0.1% FA [formic acid], 100% ACN [acetonitrile]) mixed into solvent A (0.1% FA, 100% water) over 20 minutes, resulting in a 24-minute total run time. The mass spectrometer was operated in DIA mode with MS1 scanning and MS2 precursor isolation windows between 380 and 980 m/z. MS1 scans were performed in the Orbitrap detector at 240 000 R every 0.6 s with a 5 ms ion injection time or 500% AGC (500 000 ion) target. Two-hundred fixed window MS2 DIA scans were collected at the Astral detector per cycle with 3 Th precursor isolation windows, 25% normalized collision energy, and 5 ms ion injection times with a 500% (50 000 ion) active gain control maximum. MS2 scans were collected from 150 to 2000 m/z.

DIA data were processed using Proteograph Analysis Suite. Raw MS data were processed using the DIA-NN (data-independent acquisition by neural networks) search engine (version 1.8.1) in library-free mode searching MS/ms spectra against an in silico generated spectral library of human protein entries (UP000005640_9606). Library-free search parameters include trypsin protease, 1 missed cleavage, N-terminal metformin excision, fixed modification of Cys (cysteine) carbamidomethylation, no metformin oxidation, peptide length of 7 to 30 amino acids, precursor range of 300 to 1800 m/z, and fragment ion range of 200 to 1800 m/z. MS1 and MS2 mass accuracy were set to 10 ppm. Precursor and Protein Group FDR (false discovery rate) thresholds were set at 1%. Quantification was performed on summed abundances of all unique peptides, considering only precursors passing the FDR thresholds. Given that one sample (senescent cells treated with rapamycin) had a significantly lower peptide count and also deviated from the rest of the group based on principal component analysis, the sample was eliminated from any downstream analysis. Significance was established using the Benjamini-Hochberg multiple test correction model; *P*<0.05 was considered significant. Proteomic data are available in Table S3.

### Statistics

Data are represented as mean±SD. Significance was determined using 1- or 2-way ANOVA with individual comparisons via Tukey honestly significant difference when appropriate. *P*<0.05 was considered significant.

## Results

### Senescent VSMCs Are Less Susceptible to Foam Cell Formation

**Figure 1.**
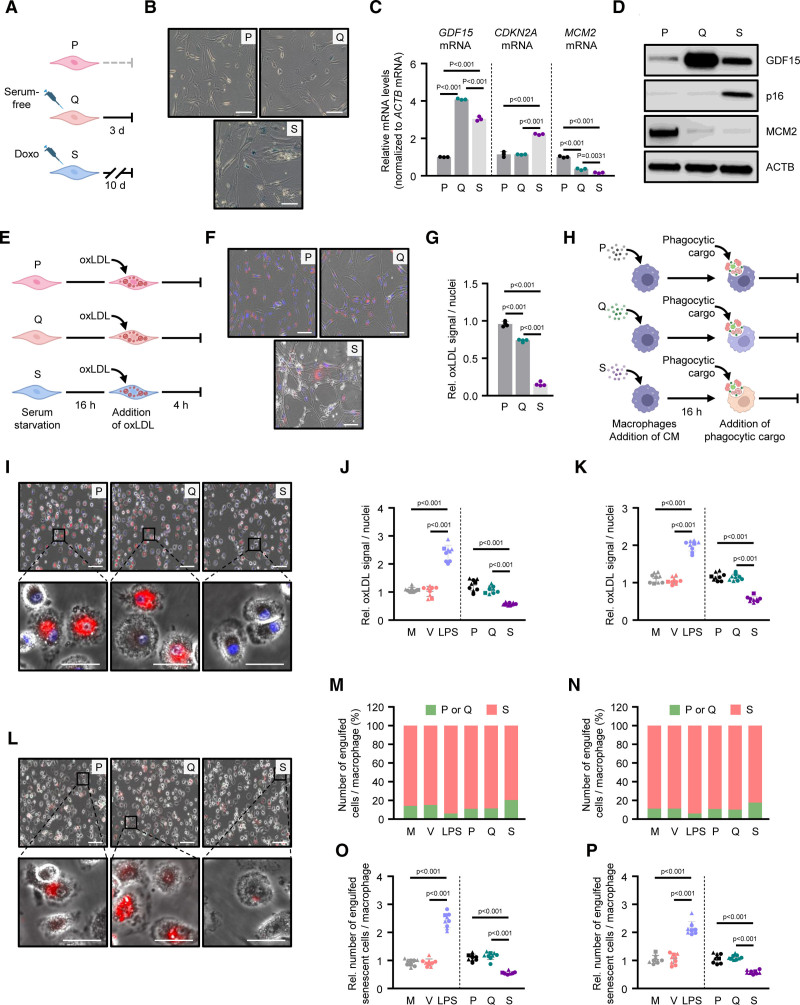
**Foam cell formation is suppressed in senescent (S) vascular smooth muscle cells (VSMCs) and macrophages. A**, Schematic showing the establishment of each VSMC state: proliferating (P), quiescent (Q), and doxorubicin-treated (S). **B**, Representative images of SA-β-Gal (senescence-associated β-galactosidase activity) for P, Q, and S VSMCs. Original magnification, ×20. **C**, Reverse transcription followed by quantitative polymerase chain reaction analysis of the levels of growth differentiation factor 15 (*GDF15*), cyclin-dependent kinase inhibitor 2A (*CDKN2A*), and minichromosome maintenance complex component 2 (*MCM2*) mRNAs in P, Q, and S VSMCs; mRNA levels were normalized to β-actin (*ACTB*) mRNA levels. **D**, Western blot analysis of the levels of proteins GDF15, CDKN2A, and MCM2 in P, Q, and S VSMCs; ACTB was used as a loading control. **E**, Schematic showing the assessment of oxLDL (oxidized low-density lipoprotein) uptake by each VSMC state. **F**, Representative images of oxLDL uptake for P, Q, and S VSMCs. Original magnification, ×20. Individual images of each channel are presented in Figure S1B. **G**, Quantification of oxLDL uptake in P, Q, and S VSMCs. The relative signal of oxLDL (red) was normalized to the number of nuclei (blue, DAPI [4′,6-diamidino-2-phenylindole]). **H**, Schematic showing the treatment of macrophages with conditioned media (CM) from P, Q, and S VSMCs (16 hours), and assessment of phagocytosis, oxLDL uptake, and efferocytosis after the addition of phagocytic cargo (Green Zymosan particles, oxLDL, and labeled VSMCs, respectively). **I**, Representative images of oxLDL uptake by macrophages (female donor) treated with P, Q, and S VSMC CM. Original magnification, ×20. The bottom row shows a magnified section of the merged image. Individual images of each channel are presented in Figure S2B. **J** and **K**, Quantification of oxLDL uptake in macrophages from female (**J**) or male (**K**) donors treated as described in (**H**) and Figure S2A. The relative signal of oxLDL (red) was normalized to the number of nuclei (blue, DAPI). **L**, Representative images of efferocytosis by macrophages (female donor) treated with P, Q, and S VSMC CM. Original magnification, ×20. The bottom row shows a magnified section of the merged image. Individual images of each channel are presented in Figure S4B. **M** and **N**, Percentage of engulfed P or Q (green) or S (red) VSMCs by macrophages in female (**M**) or male (**N**) donors treated as described in H and Figure S4A. **O** and **P**, Quantification of engulfed S VSMCs by macrophages from female (**O**) or male (**P**) donors treated as described in **H** and Figure S4A. The relative number of engulfed S cells (red) was normalized to the number of macrophage cells. Data represent the mean values ±SD from n=3 or n=4 biological replicates (experiments on the VSMCs) or n=3 different donors for each sex (>60 years old, experiments on macrophages). For graphs in **C**, **G**, **J**, **K**, **O**, and **P**, significance was established using 1-way ANOVA followed by Tukey post hoc test. LPS indicates lipopolysaccharide; M, macrophage media alone; and V, a mixture of VSMC and macrophage media.

We first established the protocols for each VSMC state: proliferating, quiescent, and senescent. Cells were plated and left untreated to generate proliferating VSMCs. We performed a 3-day serum starvation to establish quiescent VSMCs, the most common state of these cells in the arterial media. To establish senescent VSMCs, we treated proliferating VSMCs with doxorubicin, a chemotherapeutic agent that causes direct DNA damage in cycling cells, for 10 days (Figure 1A). We then performed SA-β-Gal staining on all 3 VSMC conditions and confirmed that the senescent VSMCs had increased enzymatic lysosomal activity compared with proliferating and quiescent cells (Figure 1B; Figure S1A). To properly validate the senescence phenotype, we evaluated the levels of known senescence markers by reverse transcription followed by quantitative polymerase chain reaction and western blot analyses. As expected, the levels of a canonical protein hallmark of cellular senescence, CDKN2A (p16), and the encoding transcript (*p16* mRNA), were elevated in senescent VSMCs compared with proliferating and quiescent cells (Figure 1C and 1D). Conversely, the levels of *MCM2* mRNA and MCM2 were lower in senescent VSMCs, which is known to control DNA replication and repair and is suppressed in senescence,^12^ though these were also reduced in quiescent VSMCs to a lesser degree (Figure 1C and 1D). Interestingly, the levels of *GDF15* mRNA and GDF15 were increased in senescent VSMCs, as many have shown^13^; however, quiescent VSMCs express the highest level of *GDF15* mRNA and GDF15, suggesting its expression is shared among these cell states, though it may be regulated differently (Figure 1C and 1D).

Next, we investigated the potential of different VSMC states to become foam-like cells during atherosclerosis by scavenging oxLDL. We serum-starved all cells for 16 hours before treatment with a fluorescent dye, 1,1′-dioctadecyl-3,3,3′,3′-tetramethylindocarbocyanine perchlorate to label oxLDL, and imaged the cells 4 hours later. Quantification of fluorescent signal per nuclei indicated that proliferating VSMCs ingested the most oxLDL, and quiescent VSMCs also readily took up oxLDL, although slightly less than proliferating cells (Figure 1E through 1G; Figure S1B). Interestingly, senescent VSMCs had the lowest uptake of oxLDL, a surprising finding given their pathogenic nature (Figure 1F and 1G). These results indicate that senescent VSMCs contribute to atherosclerosis, possibly through mechanisms independent of oxLDL uptake and foam cell formation. Previous studies have suggested that senescent VSMCs contribute to the secretion of hemostatic and ECM (extracellular matrix)–modifying factors and alter ECM organization, supporting the notion that these cells are not critical scavengers of oxLDL but instrumental in arterial remodeling.^4^ Senescent VSMCs may also reduce oxLDL uptake capabilities as a protective mechanism to remain viable or prevent further cellular damage, though this should be investigated further.^14,15^

### Senescent VSMC CM Reduces Macrophage Foam Cell Formation

Macrophages in the atherosclerotic niche are often primed to scavenge oxLDL.^16^ Therefore, we set out to determine the effect of VSMC-secreted proteins on macrophage foam cell development. To do so, we differentiated 6 sets of human primary monocytes from men and women over 60 years old (n=3 each) with a 7-day treatment of GM-CSF to generate proinflammatory M1-type macrophages. We chose to test monocyte-derived macrophages from older people to gain insight into molecular and functional changes possibly linked to age-associated atherosclerosis. We treated these cells with CM from proliferating, quiescent, and senescent VSMCs for 16 hours (Figure 1H). Control conditions also included macrophage media alone, a mixture of VSMC and macrophage media (1:3 dilution), and lipopolysaccharide stimulation in macrophage media (lipopolysaccharide), also administered for 16 hours (Figure S2A). All cells were then treated with 1,1’-dioctadecyl-3,3,3’,3’-tetramethylindocarbocyanine perchlorate–oxLDL and imaged 4 hours later (Figure 1I; Figure S2A through S2C). Quantification of fluorescent signal per nuclei revealed that lipopolysaccharide strongly stimulated oxLDL internalization independent of CM, while CM from senescent VSMCs appeared to strongly reduce macrophage oxLDL uptake compared with the uptake observed after treating with CM from proliferating and quiescent VSMCs in both females and males (Figure 1J and 1K; Figure S2B and S2C). These results align with the previous finding that senescent VSMCs and the proteins they secrete prevent foam cell formation and reduce macrophages’ capacity to internalize oxLDL.

### Senescent VSMC SASP Impairs Macrophage Phagocytic Function

Beyond oxLDL, macrophages play a critical role in the phagocytosis of cellular debris in the atherosclerotic plaque area. We investigated the effect of CM from various states of VSMCs on macrophage phagocytic activity using fluorescent Green Zymosan particles, which are used to mimic cellular fragments (Figure S3). Macrophage differentiation and treatment conditions were as previously described (Figure 1H; Figure S2A), but in this experiment, macrophages were given Green Zymosan particles and imaged 1 hour later (Figure S3A). Fluorescent signal per nuclei demonstrated that lipopolysaccharide treatment successfully facilitates macrophage phagocytosis, but that senescent CM reduces phagocytic activity compared with proliferating and quiescent CM in both men and women (Figure S3B through S3E). Macrophage and a mixture of macrophage and VSMC media had no impact on macrophage phagocytic activity, again indicating these findings are driven by the unique secreted proteins from senescent VSMCs (Figure S3D and S3E). Together, these data support the conclusion that the SASP of senescent VSMCs tempers macrophage phagocytic activity towards both oxLDL and cellular debris.

### SASP Inhibits Macrophage-Mediated Clearance of Senescent VSMCs

Lastly, we sought to determine whether secreted proteins from various VSMC states impacted the ability of macrophages to clear cells in an efferocytosis-like mechanism. In this case, we asked whether senescent VSMCs can evade immune surveillance by macrophages due to their SASP compared with proliferating and quiescent VSMCs. As previously described, monocytes isolated from males and females were differentiated into macrophages, and CM or controls were added as indicated (Figure 1H; Figure S4A). In this assay, proliferating and quiescent VSMCs were stained with green, fluorescent dye, while senescent VSMCs were labeled with red, fluorescent dye. Macrophages of the different treatment conditions were then fed a mixture of proliferating and senescent cells, or quiescent and senescent cells, as shown (Figure S4A). Importantly, we demonstrated that macrophages preferentially engulf senescent cells compared with proliferating or quiescent cells, regardless of the condition (Figure 1L through 1N; Figure S4B and S4C). Furthermore, lipopolysaccharide stimulation increases senescent VSMC clearance by macrophages, whereas treatment with CM from senescent VSMCs reduces the ability of macrophages from male and female donors to clear these cells (Figure 1O and 1P; Figure S4B and S4C). Treatment of macrophages with CM from proliferating or quiescent VSMCs did not alter their ability to clear senescent VSMCs, suggesting the unique SASP components derived from senescent VSMCs are responsible for the suppression in macrophage clearance (Figure 1O and 1P; Figure S4B and S4C ).

Although efferocytosis typically involves the engulfment of dead or dying cells, this mechanism may also apply to damaged cells or cells presenting as damaged due to their morphology or membrane proteins.^13,17^ Senescent cells likely appear dangerous or damaged to M1-type macrophages, which promotes their clearance, as shown here. Therefore, it is crucial to understand how senescent VSMCs interact with macrophages in atherosclerotic conditions. In aggregate, the data suggest that the SASP from senescent VSMCs inhibits macrophage efferocytotic mechanisms aimed at clearing or removing them, thereby preventing the elimination of senescent VSMCs.

### Senomorphic Treatment Partially Restores Macrophage Functions Impaired by VSMC SASP

**Figure 2.**
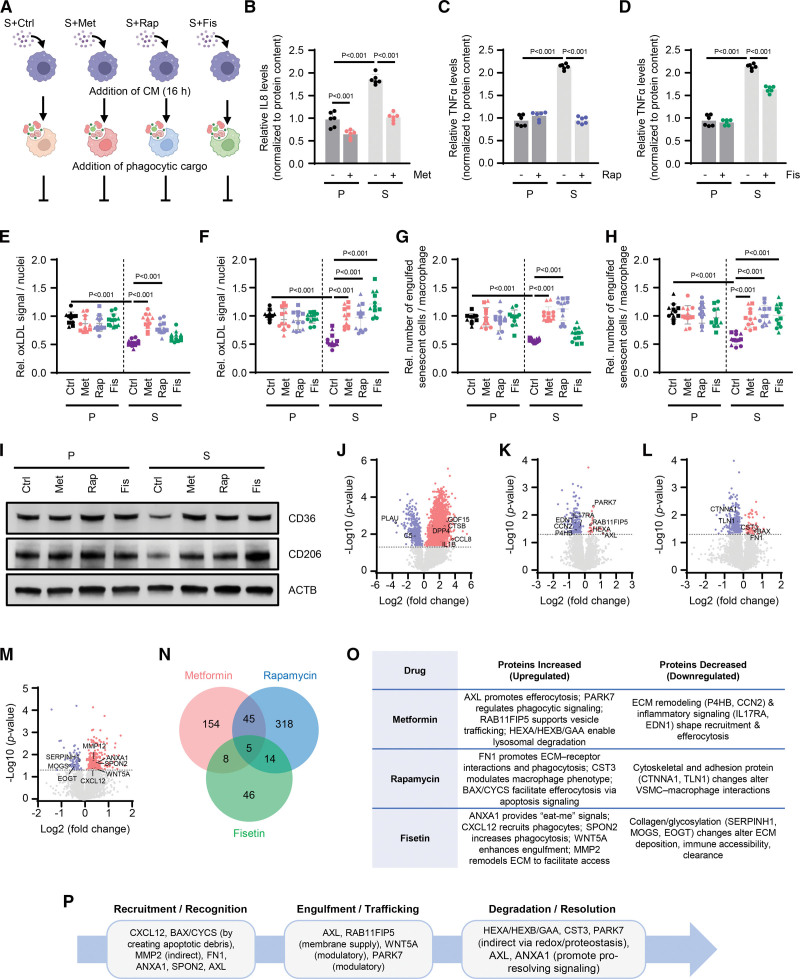
**Conditioned media (CM) from senomorphic-treated vascular smooth muscle cells (VSMCs) restore oxLDL (oxidized low-density lipoprotein) uptake and clearance of senescent (S) VSMCs by macrophages. A**, Schematic showing the treatment of macrophages with CM from S VSMCs treated with control vehicle (S+Ctrl), metformin (S+Met), rapamycin (S+Rap), and fisetin (S+Fis), and subsequent assessment of oxLDL uptake as well as efferocytosis after the addition of phagocytic cargo (oxLDL and labeled proliferating (P) or (S) VSMCs, respectively). **B**, Relative IL (interleukin) 8 secretion in P and S VSMCs treated with or without Met (10 mM). **C**, Relative TNF-α (tumor necrosis factor-α) secretion in P and S VSMCs treated with or without Rap (100 nM). **D**, Relative TNF-α secretion in P and S VSMCs treated with or without Fis (1 μM). For **B** through **D**, P and S VSMCs were treated as described in Figure S5A. **E** and **F**, Quantification of oxLDL uptake in macrophages from female (**E**) or male (**F**) donors treated as described in **A** and Figure S6A. The relative signal of oxLDL (red) was normalized to the number of nuclei (blue, DAPI [4′,6-diamidino-2-phenylindole]). **G** and **H**, Quantification of engulfed S VSMCs by macrophages from female (**G**) or male (**H**) donors treated as described in **A** and Figure S7A. The relative number of engulfed S cells (red) was normalized to the number of macrophage cells. **I**, Western blot analysis of CD36 and CD206 protein levels in macrophages (female donors) treated as described in (**A**) using P or S VSMC CM; ACTB (β-actin) was used as a loading control. **J** through **M**, Volcano plots showing the differentially secreted proteins in S compared with P VSMCs (**J**), and S VSMCs treated with Met (**K**), Rap (**L**), Fis (**M**), or control vehicle (dimethyl sulfoxide). Proteins in red (increased secretion) or blue (decreased secretion) are statistically significant. **N**, Venn diagram showing the number of secreted proteins significantly affected by each treatment and shared between different groups. **O**, Table summarizing proteins with increased or decreased secretion due to senomorphic drug treatment that may have a prominent role in efferocytosis. **P**, A schematic illustrating the 3 steps of efferocytosis, highlighting secreted proteins, which have a reported role in efferocytosis and were increased in our mass spectrometry. Data represent the mean values ±SD from n=3 different donors for each sex (> 60 years old). For graphs in **B** through **H**, significance was established using 2-way ANOVA followed by Tukey post hoc test. For the mass spectrometry analysis (**J** through **M**), significance was established using the Benjamini-Hochberg multiple test correction model, and *P*<0.05 was considered significant. ANXA1 indicates annexin A1; AXL, tyrosine-protein kinase receptor UFO; BAX, BCL2-associated X protein; C5, complement C5; C5B, complement C5B; CCL8, C-C motif chemokine ligand 8; CCN2, cellular communication network factor 2; CST3, cystatin C; CTNNA, catenin alpha 1; CXCL, stromal cell-derived factor; CYCS, cytochrome c; DPP4, dipeptidyl peptidase-4; ECM, extracellular matrix; EDN, eosinophil-derived neurotoxin; EOGT, EGF domain-specific O-linked N-acetylglucosamine transferase; FN1, fibronectin 1; GAA, acid alpha-glucosidase; GDF15, growth differentiation factor 15; HEXA, beta-hexosaminidase subunit alpha; HEXB, beta-hexosaminidase subunit beta; IL17RA, interleukin-17 receptor A; IL1B, interleukin 1 beta; MMP2, matrix metalloproteinase-2; MOGS, myelin oligodendrocyte glycoprotein; P4HB, prolyl 4-hydroxylase subunit B; PARK7, Parkinson disease protein 7; PLAU, plasminogen activator, urokinase; RAB11FIP5, Rab11-interacting protein 5; SERPINH, serpin family H member; SPON2, spondin-2; TLN, Talin; and WNT5A, Wnt family member 5A.

Due to the damaging effects of SASP, a significant area of drug research focuses on developing therapeutics to target and eliminate these cells. Senolytics are a type of drug that selectively induce apoptosis in senescent cells by inhibiting cell survival pathways like the Bcl2 (B-cell lymphoma 2) protein family.^18^ Although successful in preclinical studies, senolytics have not yet gained approval for clinical use due to issues with specificity and off-target effects.^7^ Alternatively, senomorphics aim to suppress the SASP, reducing the damage caused by senescent cells. In this study, we used drugs known for their senomorphic properties: metformin, rapamycin, and fisetin. Metformin is a clinically prescribed antidiabetic medication that has recently been shown to have anti-aging properties and suppresses the SASP.^19–21^ Rapamycin is an inhibitor of mTOR, the master regulator of cell growth and metabolism, with documented ability to extend lifespan in mice as well as diminish age-related diseases.^22,23^ Fisetin is a natural flavonoid found in fruits and vegetables such as strawberries.^24^ Among its many known benefits, fisetin has improved arterial stiffening in aging mice, demonstrating strong senolytic and senomorphic properties.^25,26^ We treated proliferating and senescent VSMCs with these drugs or a control vehicle (Figure S5A). Before conducting CM transfer experiments (Figure 2A), we measured SASP factors via ELISA in both cell states, with and without treatment. To validate metformin’s efficacy, we specifically measured IL8 levels, a SASP factor decreased by the drug, and the phosphorylation of NF-κB. Metformin effectively lowered NF-κB phosphorylation (Figure S5B) and IL8 secretion (Figure 2B) in both proliferating and senescent VSMCs; notably, senescent cells showed higher IL8 levels, which metformin reduced to nearly proliferating cell levels (Figure 2B). Similar results were seen with rapamycin and fisetin, where rapamycin significantly decreased TNF-α levels in senescent VSMCs (Figure 2C), and fisetin also reduced TNF-α but to a lesser extent (Figure 2D). For rapamycin, we also validated that treatment with the drug reduced mTOR phosphorylation (Figure S5C). Fisetin treatment often exhibits anti-inflammatory and antioxidative effects, rendering a single pathway less informative, though we measured Akt phosphorylation due to its involvement in cell survival; however, we observed no strong differences (Figure S5D).

We then performed CM transfer experiments using VSMC media from various senomorphic treatments on human macrophages from male and female donors over 60 years old (Figure S6A). As previously described, we assessed the ability of macrophages to uptake oxLDL and their efficiency in efferocytosis. As shown, senescent VSMC CM suppresses macrophage uptake of oxLDL, but interestingly, CM from senescent VSMCs treated with metformin or rapamycin significantly restored macrophage oxLDL uptake capacity in female donors (Figure 2E; Figure S6B), and all 3 drugs, metformin, rapamycin, and fisetin, rescued the oxLDL uptake in male donors (Figure 2F; Figure S6C). The influence of senomorphics on efferocytosis was similar to that of oxLDL uptake, in the case of female donors, nearly completely rescuing the ability of macrophages to engulf senescent VSMCs in the cases of metformin and rapamycin, with an insignificant trend in fisetin (Figure 2G; Figure S7A and S7B). Male donors benefited from all 3 senomorphics, significantly increasing efferocytosis of macrophages treated with senomorphic-treated senescent VSMCs compared with those treated with senescent VSMC CM (Figure 2H; Figure S7C).

### Direct and Indirect Mechanisms Facilitating Senomorphic Rescue of Macrophage Function

To dissect the manner in which senomorphics improve macrophage uptake of oxLDL and efferocytosis, we measured the corresponding protein levels of the receptors that govern these processes, CD36 and CD206. CD206, otherwise known as the mannose receptor, expressed on certain M2 macrophages, recognizes and binds mannose on the surface of apoptotic cells to promote efferocytosis.^27^ CD36 is a class B scavenger receptor that binds and internalizes oxLDL, facilitating internal lipid accumulation and foam cell formation.^28^ Here, we demonstrate that macrophages treated with CM from senescent VSMCs express lower levels of CD206 and CD36 compared with CM from proliferating VSMCs in both female (Figure 2I; Figure S8A and S8B) and male (Figure S8C through S8E) donors. Surprisingly, treatment with all 3 senomorphics, metformin, rapamycin, and fisetin, rescued CD36 and CD206 protein levels or prevented their loss in macrophages treated with CM from senescent VSMCs (Figure 2I; Figure S8A through S8E).

To better understand how the SASP from senescent VSMCs impairs macrophage function, we conducted unbiased nanoparticle-based mass spectrometry analysis of CM from proliferating and senescent VSMCs, as well as from those treated with metformin, rapamycin, and fisetin (Table S3; Figure S8F and S8G). Compared with proliferating CM, we identified 3307 proteins that were differentially secreted (*P*<0.05), including known VSMC SASP factors like GDF15, DPP4 (dipeptidyl peptidase-4), and CCL8 (C-C motif chemokine ligand 8; Figure 2J).^4,29,30^ We then aimed to pinpoint SASP proteins that are strongly secreted by senescent VSMCs and significantly diminished by senomorphic treatments that may mediate phagocytosis or efferocytosis pathways. Both metformin and rapamycin profoundly changed the secreted protein profiles of senescent VSMCs, as shown by the volcano plots (Figure 2K and 2L). Fisetin had a milder effect, indicated by a smaller shift in secreted proteins (Figure 2M). The Venn diagram illustrates the number of proteins significantly affected by each treatment and highlights the shared proteins between the different groups (Figure 2N).

A comparison of proteins significantly increased by senescent VSMCs and reduced by senomorphic treatment suggested that each senomorphic employs a distinct mechanism to restore macrophage function. The 5 shared SASP proteins, P4HA1 (prolyl 4-hydroxylase subunit alpha 1), TPP2 (tripeptidyl peptidase II), ASL (argininosuccinate lyase), CCDC134 (coiled-coil domain-containing protein 134), and OXCT1 (3-oxoacid coenzyme A transferase 1), do not indicate a common pathway related to phagocytosis or efferocytosis (Figure 2N). Pathway analysis of each drug versus senescent CM revealed proteins and pathways with potential direct and indirect effects on macrophage phagocytic activity (Figure 2O). Metformin reduced SASP proteins linked to ECM remodeling and inflammatory signals, which may impact macrophage recruitment and efferocytosis efficiency.^31,32^ Rapamycin treatment decreased proteins involved in cytoskeletal functions, such as reorganization and adhesion, potentially influencing VSMC-macrophage interactions.^33,34^ Fisetin lowered SASP proteins regulating ER stress and collagen chaperones, among others, possibly affecting eat-me signals or ECM properties to influence cell clearance indirectly.^35,36^ The SASP proteins decreased by senomorphics seem to mostly exert indirect effects on phagocytosis and efferocytosis, prompting us to investigate those proteins whose levels were increased by these drugs.

Overall, fewer proteins were significantly increased after senomorphic treatment compared with decreased; however, among the proteins increased, a larger proportion was linked to phagocytosis and efferocytosis. Proteins notably increased by metformin include AXL (tyrosine-protein kinase receptor UFO), PARK7 (Parkinson disease protein 7), RAB11FIP5 (Rab11-interacting protein 5), HEXA (beta-hexosaminidase subunit alpha), HEXB (beta-hexosaminidase subunit beta), and GAA (acid alpha-glucosidase), all known to participate in various stages of phagocytosis. AXL enhances efferocytosis,^37^ PARK7 modulates phagocytosis,^38^ RAB11FIP5 supports recruitment of phagocytic machinery,^39^ and HEXA, HEXB, and GAA collectively facilitate lysosomal breakdown of phagosomes.^40–42^ Rapamycin treatment elevated several proteins, FN1 (fibronectin 1), CST3 (cystatin C), BAX (BCL2-associated X protein), and CYCS (cytochrome c) in the CM from senescent VSMCs, all associated with efferocytosis. Among the key pathways enriched, ECM-receptor interactions stood out, highlighted by FN1, which has been linked to phagocytosis.^43,44^ CST3 has the potential to promote apoptosis as well as influence macrophage phenotype,^45^ while BAX and CYCS affect efferocytosis indirectly through their roles in apoptosis.^46,47^ In addition, fisetin increased levels of ANXA1 (annexin A1), CXCL12 (stromal cell–derived factor 1), SPON2 (spondin-2), WNT5A (Wnt family member 5A), and MMP2 (matrix metalloproteinase-2). ANXA1 encodes an eat-me signal that promotes efferocytosis directly.^48^ The chemokine CXCL12 facilitates efferocytosis by recruiting phagocytes,^49^ and SPON2, an extracellular matrix protein, acts as an opsonin for phagocytosis.^50^ WNT5A promotes phagocytosis,^51^ and MMP2 may indirectly enhance macrophage access to targets via ECM remodeling, although this effect is often context-dependent.^52^ These findings suggest that modifying the SASP from senescent VSMCs can influence all 3 key steps in efferocytosis: recruitment, engulfment, and degradation (Figure 2P).^53^ Interestingly, the identified SASP factors do not intersect at one pathway or macrophage receptor, suggesting that these proteins participate in complementary stages of macrophage function. This distribution of proteins across the sequential steps of efferocytosis implies that senescent VSMCs impair macrophage function through coordinated modulation of multiple effector pathways, rather than through the action of a single SASP component. Further research is needed to elucidate the precise mechanisms by which senomorphic drugs alter the secretion of these SASP factors to improve macrophage function during the development of atherosclerosis.

## Discussion

The central finding of this study is that the SASP of VSMCs inhibits crucial macrophage functions, such as the phagocytosis of oxidized lipids and efferocytosis of senescent cells, which were restored by treatment with senomorphic drugs. Consequently, macrophages may fail to engage senescent VSMCs, allowing these cells to evade immune clearance, sustain their viability, and further propagate the SASP within the atherosclerotic niche. Numerous studies have shown that eliminating senescent cells in age-related pathologies, including atherosclerosis, improves outcomes and reduces disease severity, particularly by lessening foam cell formation.^3,54–57^ Thus, our study provides a potential mechanism by which senescent cells exacerbate atherosclerosis by impeding the macrophage functions necessary for cholesterol efflux and the clearance of apoptotic or damaged cells and debris. Our data support a model in which the functional impairment of macrophages by senescent VSMCs occurs partially through the secretion of multiple SASP factors that work together to disrupt lipid uptake, phagocytosis, and efferocytosis. The widespread distribution of proteins across pathways related to efferocytosis indicates that the suppression of macrophage function is a coordinated rather than synergistic effect, although further investigation is needed to determine the exact mechanism of action.

An atherosclerotic artery is characterized by a complex mixture of cholesterol, fat, calcium, cellular waste, dead cells, fibrin, macrophages, T cells, and smooth muscle cells, both dedifferentiated and senescent.^58^ The disease is driven by a combination of lipids and inflammation, resulting in the presence of both pro-inflammatory and anti-inflammatory molecules in the plaque area. Senescent VSMCs have been shown to secrete pro-inflammatory cytokines, such as IL6 and IL8, as part of the SASP.^59^ Recently, we discovered that senescent VSMCs also contribute a range of hemostatic factors, complement proteins, and extracellular matrix-modifying proteins to the SASP.^4,58^ Notably, many proteins identified by mass spectrometry exhibit both pro-inflammatory and anti-inflammatory properties. Recent studies have also demonstrated that SASP factors can be packaged into extracellular vesicles (EVs), further illuminating the complexity of the SASP in immune regulation. Specifically, Gluchowska et al showed that senescent VSMCs secrete EVs containing known SASP proteins SERPINF1 (serpin family F member 1) and THBS1 (thrombospondin 1), which can also impact the immune response.^13^ Consistent with this concept, our proteomic data set revealed that many immune-related proteins were influenced by senomorphics, elevating the possibility that a subset of the SASP factors we identified may be packaged and delivered by EVs to macrophages rather than soluble secreted proteins. In this study, EV-mediated protein delivery and signaling were not directly measured, though EV proteins may be captured utilizing nanoparticle methods used here^60^; however, our findings, paired with existing literature, suggest that future investigations into whether EV-mediated delivery of SASP factors represents an additional mechanism contributing to immune dysfunction during atherosclerosis are warranted. Regardless of the mode of delivery, defining the overall secretory profile of senescent VSMCs remains critical for understanding their functional impact on macrophage behavior.

To determine the secreted proteins most affected by senomorphics, we performed nanoparticle-based mass spectrometry on CM from proliferating and senescent VSMCs with and without metformin, rapamycin, and fisetin treatment (Figure 2J through 2O). Interestingly, very few differentially abundant secreted proteins were shared among the treatment groups. Still, the processes and pathways featured overlap in extracellular matrix remodeling, cell death-associated proteins, and the ability to impact various stages of phagocytosis and efferocytosis.

The ability of three mechanistically distinct senomorphic agents to restore macrophage function despite minimal overlapping of SASP proteins further reinforces a model where disruption of coordinated SASP signaling networks, rather than a single harmful factor, causes macrophage dysfunction. Importantly, this also indicates that rebalancing the overall secretory environment, instead of focusing on one protein, may represent an effective therapeutic approach. In addition, although senomorphic agents are generally recognized for their broad beneficial effects on inflammation and metabolic balance,^61^ our findings highlight a more specific and functionally relevant outcome, underscoring that such broad effects may mask cell–type–specific or context-dependent consequences that limit their precision as therapeutic interventions. In addition to these general improvements, senomorphic treatment notably maintained or rescued scavenger receptors, CD36 and CD206 expression in macrophages (Figure 2I) compared with those exposed to senescent CM alone, directly indicating enhanced macrophage phagocytic function. Together, these findings suggest that the SASP encompasses multiple mechanisms capable of impairing macrophage phagocytic function, and that reducing the secretion of these detrimental factors or increasing beneficial factors may provide new opportunities to intervene in the development of atherosclerosis.

A previous study found that senescent cells reduce macrophage efferocytosis through direct contact rather than the SASP, and that senescent fibroblasts resist macrophage clearance.^62^ Although different cell types, disease states, and monocyte differentiation methods may influence the results, it has long been proposed that macrophages play a primary role in clearing senescent cells.^63^ In our study, we show that macrophages, under our experimental conditions, preferentially clear senescent VSMCs; however, in the presence of CM containing SASP factors, this engulfment is diminished. A recent study discovered that aging adversely affects macrophage functions, including efferocytosis, thereby hindering the regression of atherosclerosis.^64^ Our findings offer a potential explanation, indicating that senescent cells, which accumulate with age and disease, release SASP factors that lead to the decreased expression of essential receptors and pathways while allowing senescent cells to evade macrophage surveillance. Because macrophage function may wane with age, we obtained human monocyte-derived macrophages from donors who were >60 years old for our study, capturing the functional effects of senescent VSMCs on macrophages from people who are most susceptible to atherosclerotic vascular diseases. While a systematic assessment of young and aged VSMC-macrophage combinations would enhance our understanding of age-dependent crosstalk, such analyses extend beyond the scope of this brief report and offer an important area of subsequent investigation. Future studies should explore the mechanisms by which senescent cells escape immune surveillance in dynamic model systems to identify new therapeutic directions for both the development and regression of atherosclerosis.

## ARTICLE INFORMATION

### Author Contributions

A.B. Herman and D. Tsitsipatis conceptualized the study and designed experiments; A.B. Herman, D. Tsitsipatis, T. Rodriguez Rivera, M. Kaileh, A.N. Okereke, A. Gupta, A. Singh, S.M. Raph, and C. Henry-Smith performed experiments and analyzed data; A.B. Herman wrote the manuscript.

### Disclosures

None.

### Supplemental Material

Tables S1–S3

Figures S1–S8

Major Resource Table

## Supplementary Material
