# Supplementary material for: Impairment of Macrophage Functions by the Senescence-Associated Secretory Phenotype of Vascular Smooth Muscle Cells—Brief Report
Source: Arterioscler Thromb Vasc Biol. 2026 Apr 2;46(5):e324299. doi: 10.1161/ATVBAHA.125.324299 (PMC13098668; doi:10.1161/ATVBAHA.125.324299)
Supplement: Supplementary file 1 [file atv-46-e324299-s001.pdf]

SUPPLEMENTAL MATERIALS

Impairment of Macrophage Functions by the Senescence-Associated Secretory Phenotype of Vascular Smooth Muscle Cells – Brief Report

Dimitrios Tsitsipatis[1], Tatiana Rodriguez Rivera[1], Mary Kaileh[2], Ada N. Okereke[1], Aditi Gupta[2], Amit Singh[2], Sean M. Raph[1], Charnae' Henry-Smith[1], Allison B. Herman[1,*]

[1]Laboratory of Cardiovascular Science, [2]Laboratory of Molecular Biology and Immunology, National Institute on Aging (NIA) Intramural Research Program (IRP), National Institutes of Health (NIH), Baltimore, MD 21224, USA

Major Resources Table

Antibodies

Target antigen	Vendor or Source	Catalog #	Working concentration	Lot # (preferred but not required)	Persistent ID / URL
CDKN2A/p16 (JC8)	Santa Cruz	sc-56330	400 ng/mL		https://www.scbt.com/p/p16-antibody-jc8
GDF-15 (G-5)	Santa Cruz	sc-377195	400 ng/mL		https://www.scbt.com/p/gdf-15-antibody-g-5
MCM2	Cell Signaling	4007	10 ng/mL		https://www.cellsignal.com/products/primary-antibodies/mcm2-antibody/4007
Phospho-NF-kappaB p65 (Ser536) (93H1)	Cell Signaling	3033	57 ng/mL		https://www.cellsignal.com/products/primary-antibodies/phospho-nf-kappab-p65-ser536-93h1-rabbit-monoclonal-antibody/3033
NF-kappaB p65 (D14E12)	Cell Signaling	8242	139 ng/ml		https://www.cellsignal.com/products/primary-antibodies/nf-kappab-p65-d14e12-rabbit-monoclonal-antibody/8242
Phospho-mTOR (Ser2481)	Cell Signaling	2974	56 ng/ml		https://www.cellsignal.com/products/primary-antibodies/phospho-mtor-ser2481-antibody/2974
mTOR (7C10)	Cell Signaling	2983	94 ng/ml		https://www.cellsignal.com/products/primary-antibodies/mtor-7c10-rabbit-monoclonal-antibody/2983
Phospho-Akt (Ser473) (D9E)	Cell Signaling	4060	91 ng/ml		https://www.cellsignal.com/products/primary-antibodies/phospho-akt-ser473-d9e-rabbit-monoclonal-antibody/4060
Akt (pan) (C67E7)	Cell Signaling	4691	35 ng/ml		https://www.cellsignal.com/products/primary-antibodies/akt-pan-c67e7-rabbit-monoclonal-antibody/4691
CD36 (EPR6573)	Abcam	ab133625	1.988 µg/mL		https://www.abcam.com/en-us/products/primary-antibodies/cd36-antibody-epr6573-ab133625
CD206	Abcam	ab133625	1 µg/mL		https://www.abcam.com/en-us/products/primary-antibodies/cd36-antibody-epr6573-ab133625
ACTB (C4)	Santa Cruz	sc-56330	400 ng/mL		https://www.scbt.com/p/beta-actin-antibody-c4

DOI [to be added]

Cultured cells

Name	Vendor or Source	Sex (F, M, or unknown)	Persistent ID / URL
Primary human coronary artery vascular smooth muscle cells	LifeLine Cell Technology (Cat. #: FC-0031)	M	https://www.lifelinecelltech.com/shop/cells/smooth-muscle-cells/coronary-smooth-muscle-cells/coronary-artery-smooth-muscle-cells-fc-0031/
Monocyte-derived macrophages	In the study		Donors' demographics are included in Table S1

Data availability and software

Description	Source / Repository	Persistent ID / URL
Mass spectrometry data	MassIVE (dataset identifier MSV000100792)	https://massive.ucsd.edu/ProteoSAFe/private-dataset.jsp?task=af7b818a979944d6887c6caa93329b01
BZ-X800 analyzer	Keyence	https://www.keyence.com/landing/microscope/lp_fluorescence.jsp
ImageJ	NIH provided	
GraphPad Prism	NIH provided	
EndNote	NIH provided	

Other

Description	Source / Repository	Persistent ID / URL
VascuLife SMC Medium Complete Kit	LifeLine Cell Technology	https://www.lifelinecelltech.com/shop/culture-media/smooth-muscle-culture-media/vasculife-smooth-muscle-cell-medium-ll-0014/
ImmunoCult™-SF Macrophage Medium	StemCell Technologies	https://www.stemcell.com/products/immunocult-sf-macrophage-medium.html
Senescence beta-Galactosidase Staining Kit	Cell Signaling	https://www.cellsignal.com/products/cellular-assay-kits/senescence-beta-galactosidase-[…]ksb2YOVCdB5SjmBZ-ZtipL8FW7Cr1x8Ik1Zhq2sKJh2_kBoCO0UQAvD_BwE
Recombinant Human GM-CSF (CHO-expressed) Protein	R&D Systems	https://www.rndsystems.com/products/recombinant-human-gm-csf-cho-expressed-protein_795[…]_BwE&gclsrc=aw.ds&gbraid=0AAAAAD_kmX2cAtdOvHQ1UQ0Ru_RdSuG6T
Human IL-8/CXCL8 Quantikine ELISA Kit	R&D Systems	https://www.rndsystems.com/products/human-il-8-cxcl8-quantikine-elisa-kit_d8000c?_gl=1[…]_BwE&gclsrc=aw.ds&gbraid=0AAAAAD_kmX2cAtdOvHQ1UQ0Ru_RdSuG6T
Human TNF-alpha Quantikine QuicKit ELISA	R&D Systems	https://www.rndsystems.com/products/human-tnf-alpha-quantikine-quickit-elisa_qk210?_gl[…]C5jMQAvD_BwE&gclsrc=aw.ds&gbraid=0AAAAAD_kmX2cAtdOvHQ1UQ0Ru_RdSuG6T
Doxorubicin	Selleckchem	https://www.selleckchem.com/products/doxorubicin-topoisomerase-inhibitor.html
Metformin Hydrochloride	Selleckchem	https://www.selleckchem.com/products/metformin-hcl.html
Rapamycin (Sirolimus)	Selleckchem	https://www.selleckchem.com/products/rapamycin-sirolimus-mtor-inhibitor.html
Fisetin	Selleckchem	https://www.selleckchem.com/products/fisetin.html
Oxidized Low-density Lipoprotein (OxLDL) and DiI-OxLDL	ThermoFisher Scientific	https://www.thermofisher.com/order/catalog/product/L34357
NucBlue™ Live ReadyProbes™ Reagent (Hoechst 33342)	ThermoFisher Scientific	https://www.thermofisher.com/order/catalog/product/R37605?SID=srch-srp-R37605

DOI [to be added]

Lipopolysaccharide (LPS) Solution (500X)	ThermoFisher Scientific	https://www.thermofisher.com/order/catalog/product/00-4976-93
PKH67 Green Fluorescent Cell Linker Kit for General Cell Membrane Labeling	Sigma-Aldrich	https://www.sigmaaldrich.com/US/en/product/sigma/pkh67gl?srsltid=AfmBOooDa2aQgbL2NqkMyu5dWUspB4avg7KEKLmW8hgV3xX_86pvneK0
PKH26 Red Fluorescent Cell Linker Kit for General Cell Membrane Labeling	Sigma-Aldrich	https://www.sigmaaldrich.com/US/en/product/sigma/pkh26gl?srsltid=AfmBOooWcpZCMVKP_7QIynxiEn36ANrQXwd5GelgSWw3_lebGqjlhKGL
Phagocytosis Assay Kit (Green Zymosan)	Abcam	https://www.abcam.com/en-us/products/assay-kits/phagocytosis-assay-kit-green-zymosan-ab234053
SYBR Green mix	Kapa Biosystems	https://www.fishersci.com/shop/products/kapa-sybr-fast-master-mix-2/501965206

DOI [to be added]

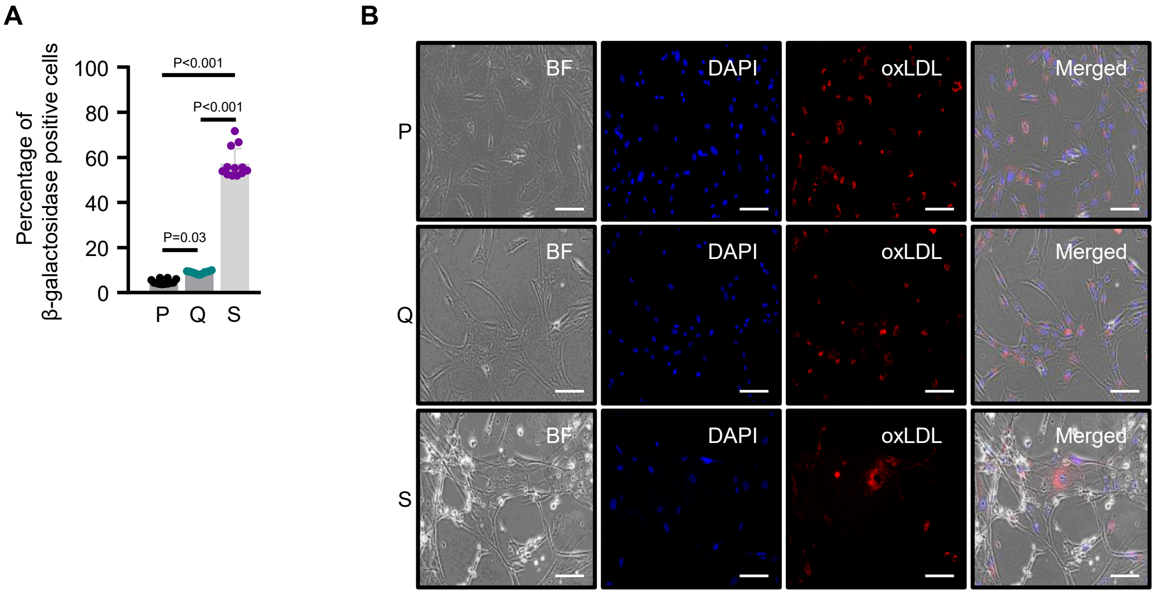

Supplemental Figure S1. Foam-cell formation is suppressed in senescent VSMCs. (A) Quantification of senescence-associated β-galactosidase (SA-β-Gal) signal in proliferating (P), quiescent (Q), and senescent (S) VSMCs; representative images can be found in Figure 1B. Per **(B)** Representative individual images of each channel for Figure 1F; 'merged' images in this panel are identical to Figure 1F. Original magnification, ×20. For graph in A, significance was established using one-way ANOVA with Tukey's post-hoc test.

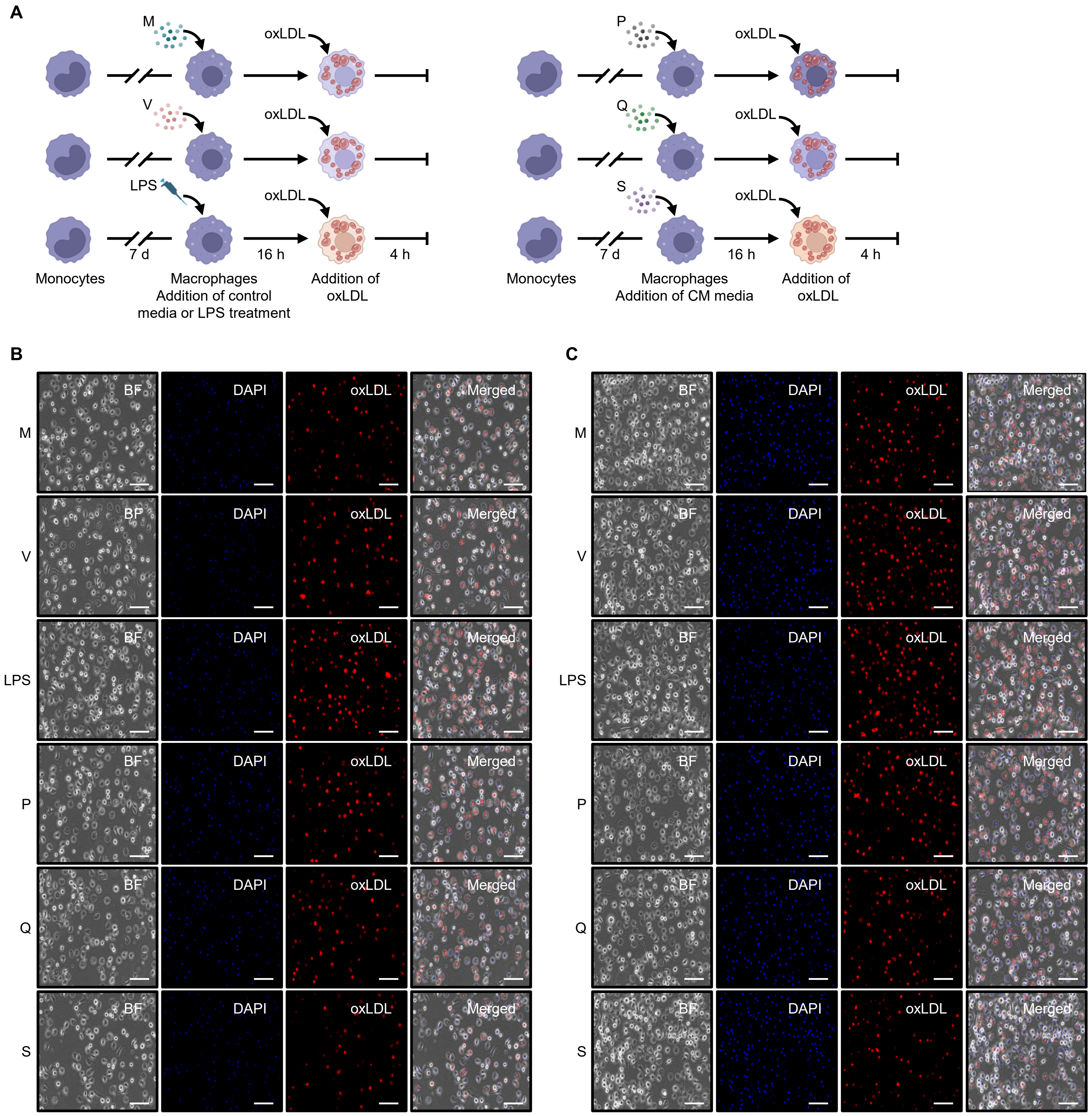

Supplemental Figure S2. Senescent VSMC conditioned media suppresses the formation of foam-like macrophages (remaining representative images for Fig. 1I-K). (A) Schematic showing the establishment of monocyte-derived macrophage cultures (7-day treatment with GM-CSF), subsequent treatment with macrophage media (M), a mixture of macrophage and VSMC media (V), LPS (LPS), proliferating (P), quiescent (Q), and senescent (S) VSMC conditioned media for 16 h, and assessment of oxLDL by macrophages. **(B, C)** Representative images of oxLDL uptake by macrophages from female (B) and male (C) donors treated as described in (A). For macrophages from a female donor (B) treated with P, Q, and S VSMC conditioned media, the 'merged' images in this panel are identical to Figure 1I. Original magnification, ×20. **(C)** Representative images of oxLDL uptake by macrophages (male donor) treated as described in (A). Original magnification, ×20.

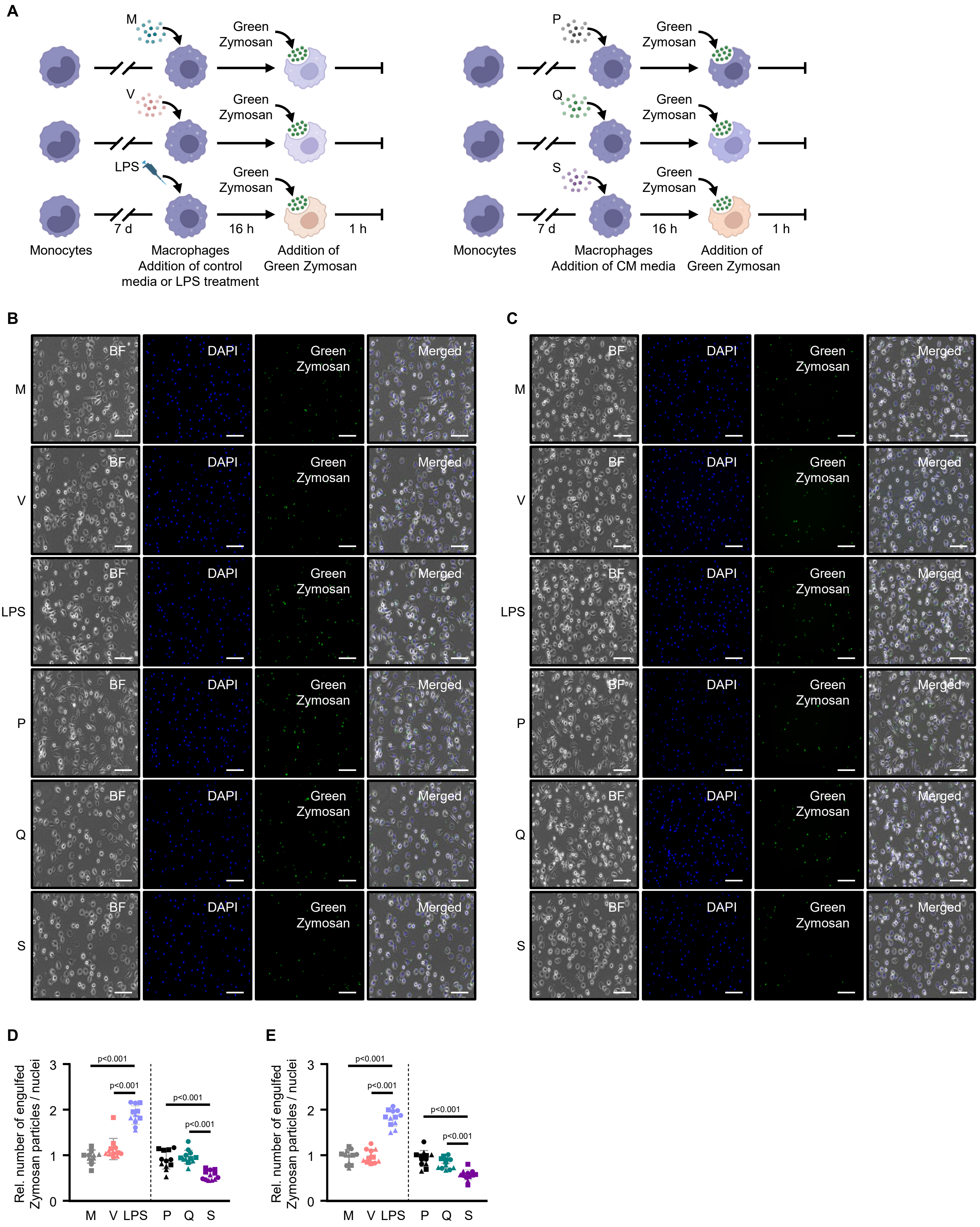

Supplemental Figure S3. Conditioned media from senescent VSMCs attenuates phagocytosis by macrophages. (A) Schematic showing the establishment of monocyte-derived macrophage cultures (7-day treatment with GM-CSF), subsequent treatment with macrophage media (M), a mixture of macrophage and VSMC media (V), LPS (LPS), proliferating (P), quiescent (Q), and senescent (S) VSMC conditioned media for 16 h, and assessment of phagocytosis using Green Zymosan particles by macrophages. **(B, C)** Representative images of phagocytosis by macrophages from female (B) and male (C) donors treated as described in (A). Original magnification, ×20. **(D, E)** Quantification of phagocytosis in macrophages from female (D) or male (E) donors treated as described in (A). The relative number of engulfed Zymosan particles (green) was normalized to the number of nuclei (blue, DAPI). Data represent the mean values ±SD from n= 3 different donors for each sex (> 60 years old). For graphs in D and E, significance was established using one-way ANOVA with Tukey's post-hoc test.

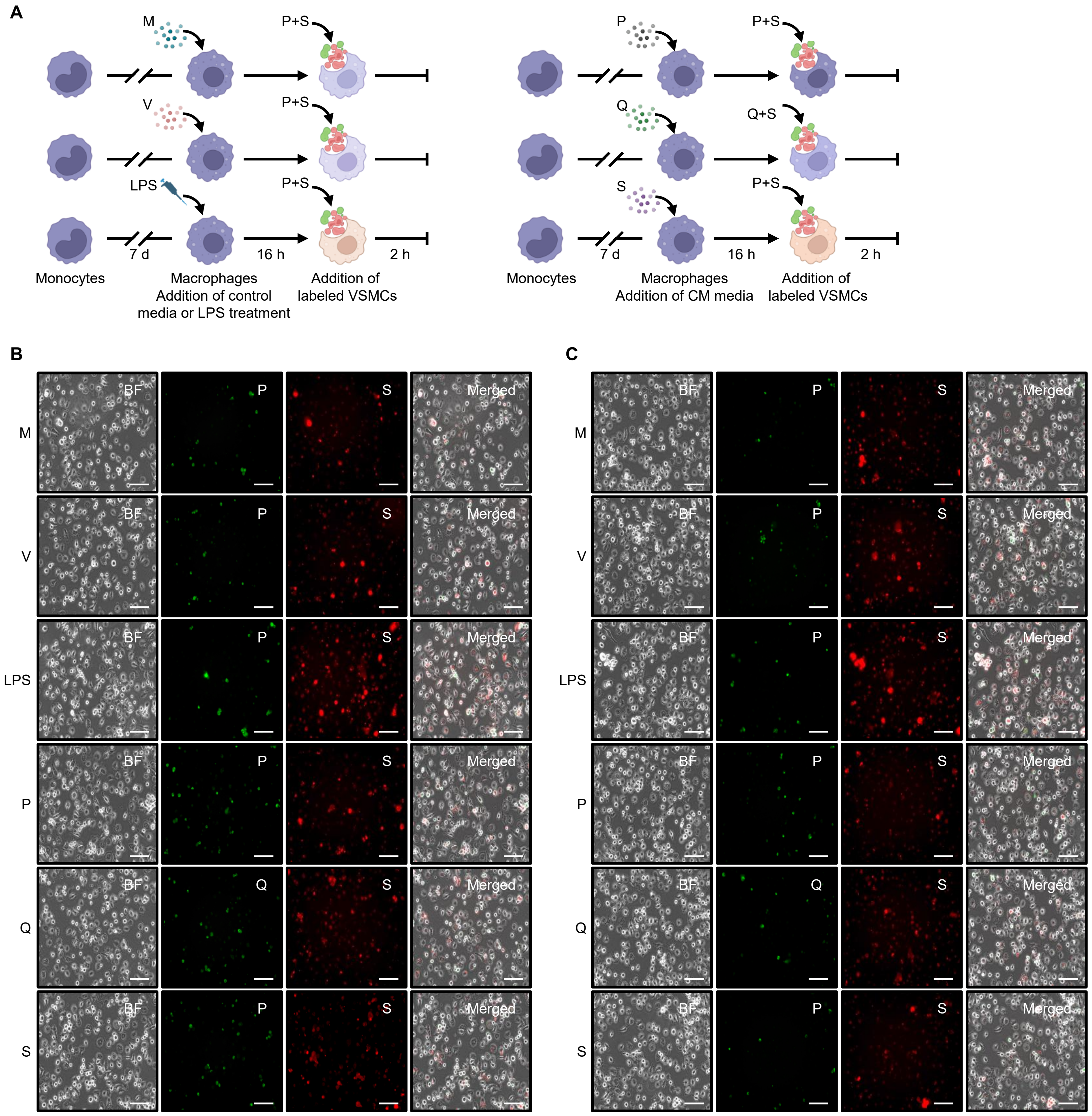

Supplemental Figure S4. Conditioned media from senescent VSMCs attenuates the engulfment of senescent VSMCs by macrophages (remaining representative images for Fig. 2L-P). (A) Schematic showing the establishment of monocyte-derived macrophage cultures (7-day treatment with GM-CSF), subsequent treatment with macrophage media (M), a mixture of macrophage and VSMC media (V), LPS (LPS), proliferating (P), quiescent (Q), and senescent (S) VSMC conditioned media for 16 h, and assessment of efferocytosis using green-labeled proliferating (or quiescent) or red-labeled senescent VSMCs. **(B, C)** Representative images of efferocytosis by macrophages from female (B) or male (C) donors treated as described in A. For macrophages from a female donor (B) treated with P, Q, and S VSMC conditioned media, the ‘merged’ images in this panel are identical to Figure 1L. Original magnification, ×20.

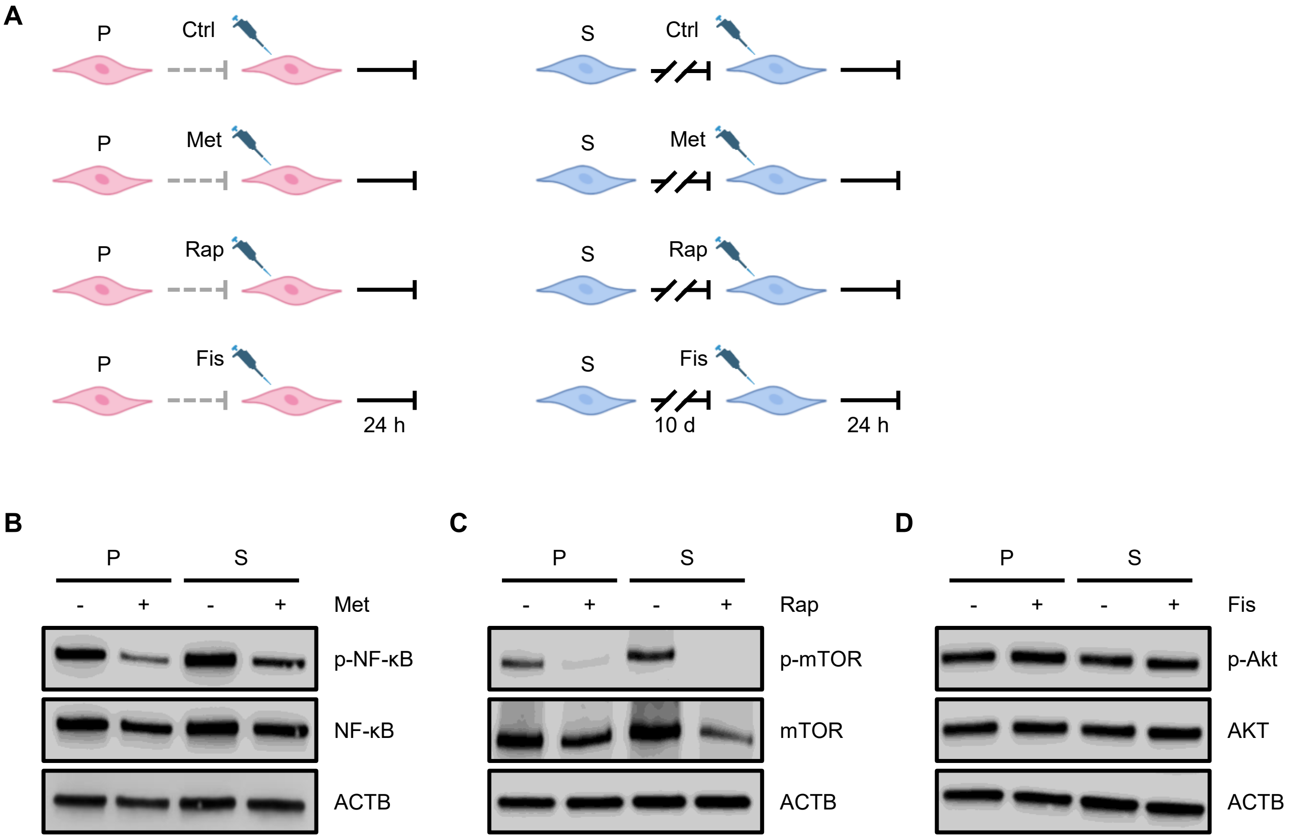

Supplemental Figure S5. Establishing the senomorphic drug treatments in proliferating and senescent VSMCs. (A) Schematic showing the establishment of a 24-h treatment with metformin (Met), rapamycin (Rap), fisetin (Fis), or control vehicle (Ctrl) in proliferating (P) and senescent (S) VSMCs. **(B)** Western blot analysis of the phosphorylation of NF-κB in P and S VSMCs treated with or without Met; NF-κB was used to assess changes in the total protein levels, whereas ACTB was used as a loading control. **(C)** Western blot analysis of the phosphorylation of mTOR in P and S VSMCs treated with or without Rap; mTOR was used to assess changes in the total protein levels, whereas ACTB was used as a loading control. **(D)** Western blot analysis of the phosphorylation of AKT in P and S VSMCs treated with or without Fis; AKT was used to assess changes in the total protein levels, whereas ACTB was used as a loading control.

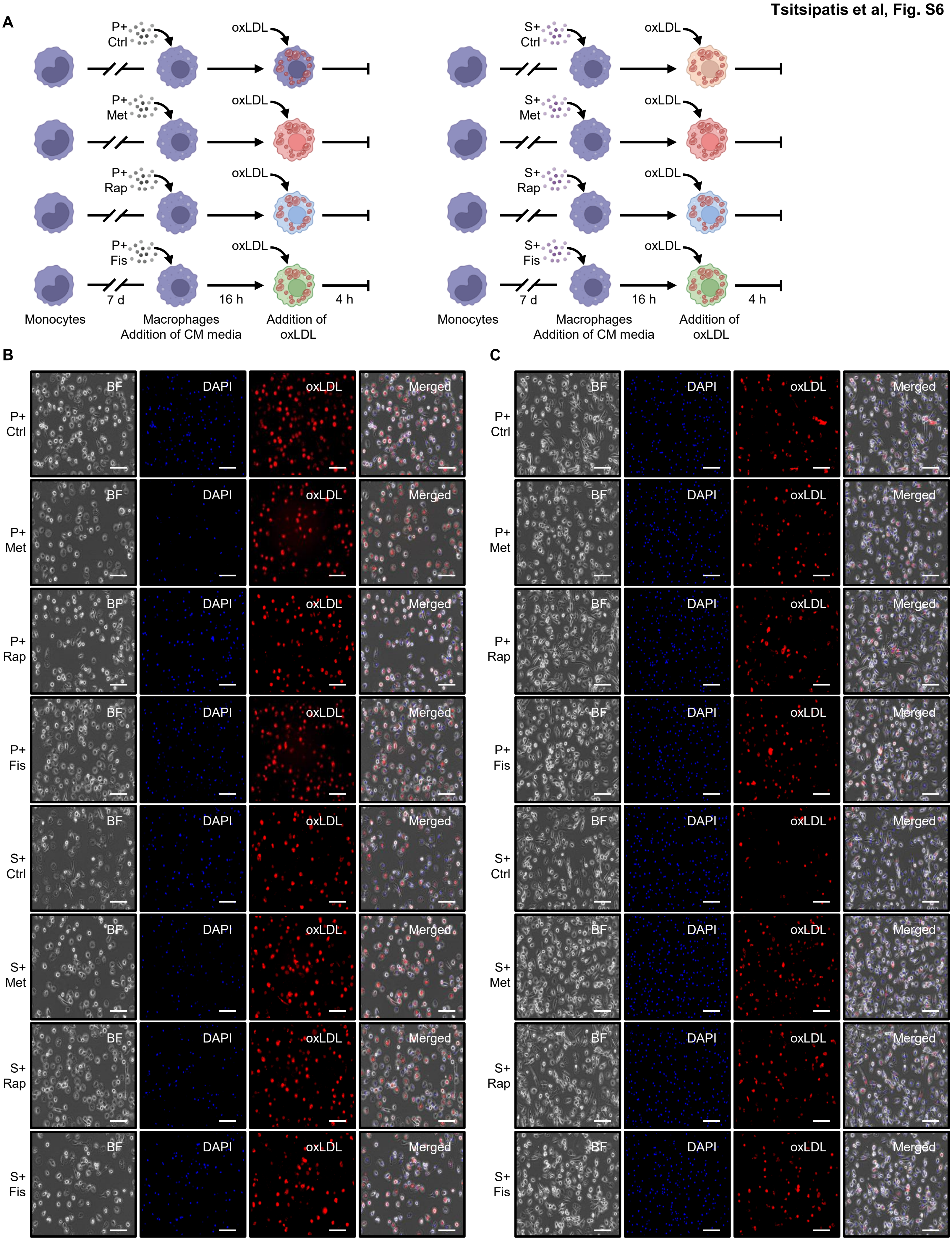

Supplemental Figure S6. Conditioned media from senomorphic-treated senescent VSMCs restores the oxLDL uptake by macrophages (representative images for Fig. 2E, F). (A) Schematic showing the establishment of monocyte-derived macrophage cultures (7-day treatment with GM-CSF), subsequent 16-h treatment with conditioned media from proliferating (P) and senescent (S) VSMCs treated with metformin (Met), rapamycin (Rap), fisetin (Met), or control vehicle (Ctrl), and assessment of oxLDL by macrophages. **(B, C)** Representative images of oxLDL uptake by macrophages derived from female (B) and male (C) donors treated as described in (A). Original magnification, ×20.

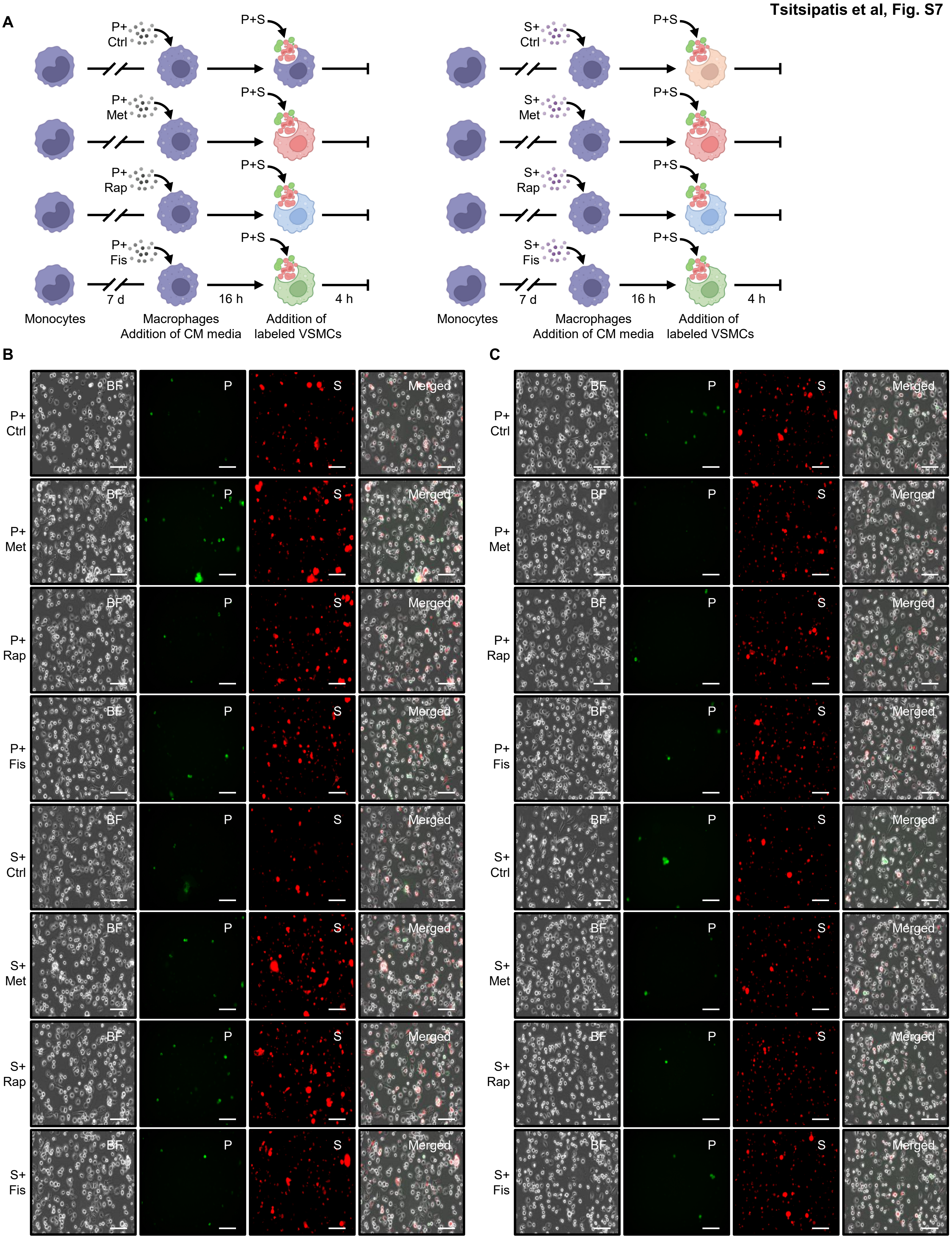

Supplemental Figure S7. Conditioned media from senomorphic-treated senescent VSMCs restores the engulfment of senescent VSMCs by macrophages (representative images for Fig. 2G, H). (A) Schematic showing the establishment of monocyte-derived macrophage cultures (7-day treatment with GM-CSF), subsequent 16-h treatment with conditioned media from proliferating (P) and senescent (S) VSMCs treated with metformin (Met), rapamycin (Rap), fisetin (Met), or control vehicle (Ctrl), and assessment of efferocytosis using green-labeled proliferating or red-labeled senescent VSMCs by macrophages. **(B, C)** Representative images of efferocytosis by macrophages from female (B) and male (C) donors treated as described in (A). Original magnification, ×20.

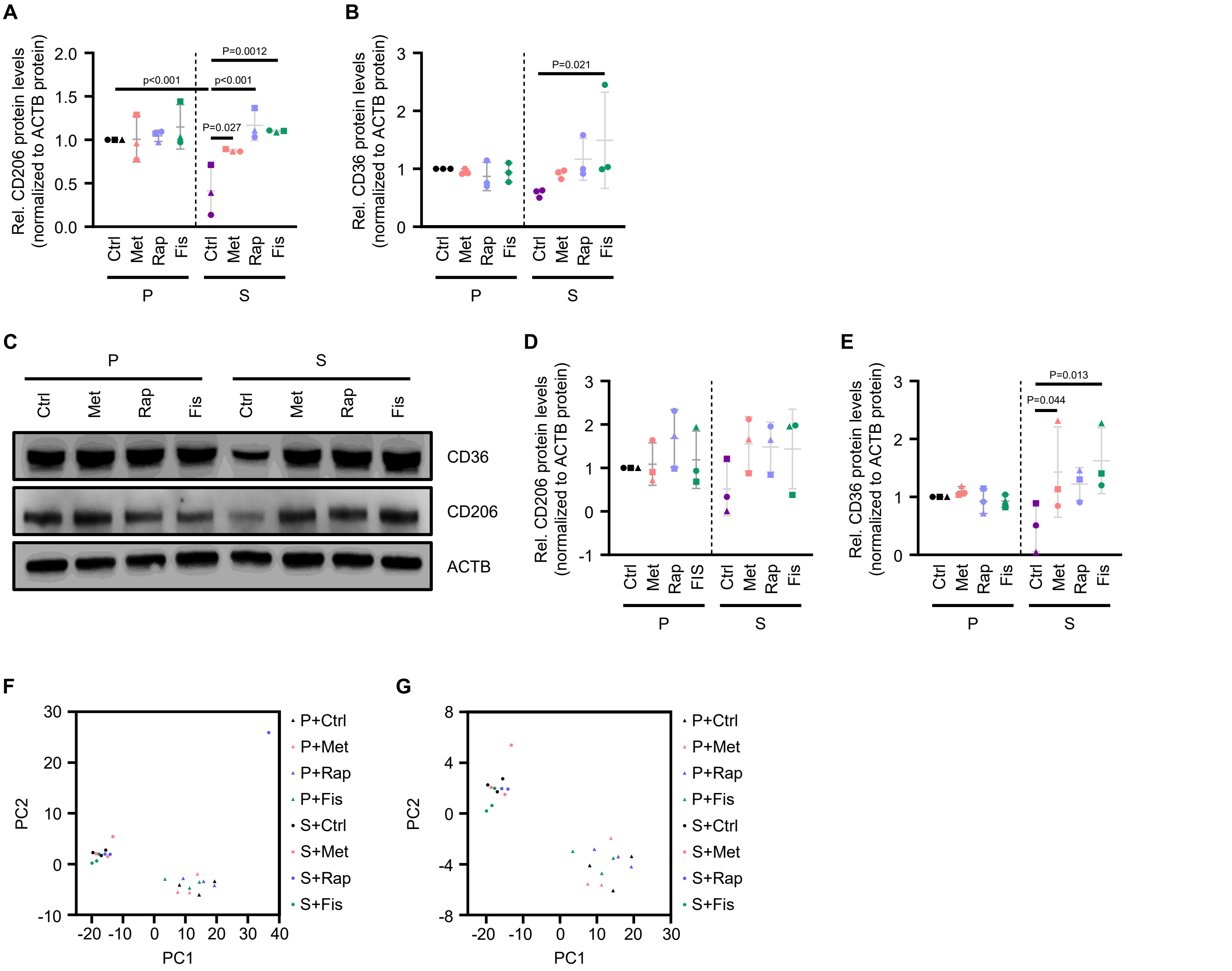

Supplemental Figure S8. Identifying prominent receptors and secreted proteins governing the senomorphic-mediated restoration of efferocytosis by macrophages. (A, B) Relative protein levels of CD36 (A) and CD206 (B) in female donors treated as described in Figure 2A. CD36 and CD206 signal levels were normalized to the ACTB signal. Representative Western blot can be found in Figure 2I. **(C-E)** Representative Western blot from a male donor treated as described in Figure 2A (C). Relative protein levels of CD36 (D) and CD206 (E) in male donors treated as described in Figure 2A. CD36 and CD206 signal levels were normalized to the ACTB signal. **(F, G)** Principal component analysis using secreted proteins differentially present in the conditioned media for proliferating and senescent VSMCs, treated as described in Figure VA in the Data Supplement. The outlier (S2_Rap) was removed from the downstream analysis (G). For graphs in A, B, D, and E, significance was established using two-way ANOVA with Tukey's post-hoc test.

Table 1.

Demographics of donors used in this study

Sex	Age (years)	Mean age (years)
Female	60	
Female	69	67 ± 6
Female	72	
Male	61	
Male	67	66 ± 4
Male	69	

Table 2.

List of primers used in this study

Oligo name	Sequence
ACTB_F	CATGTACGTTGCTATCCAGGC
ACTB_R	CTCCTTAATGTCACHCACGAT
CDKN2A_F	GTTACGGTCGGAGGCCG
CDKN2A_R	GTGAGAGTGGCGGGGTC
GDF15_F	GACCCTCAGAGTTGCACTCC
GDF15_R	GCCTGGTTAGCAGGTCCTC
MCM2_F	ATTTGGTCCTGGGTCCTTTC
MCM2_R	CGCTGGTAGTTCTGATAGATGG
